# Meaningful Activities Among Vulnerable Older Adults Living With Dementia

**DOI:** 10.1093/geroni/igaa057.3070

**Published:** 2020-12-16

**Authors:** Theresa Allison, Jennie Gubner, Alexander Smith

**Affiliations:** 1 UCSF Division of Geriatrics/San Francisco VA Medical Center, San Francisco, California, United States; 2 University of Arizona, Tucson, Arizona, United States; 3 University of California San Francisco Division of Geriatrics, San Francisco, California, United States

## Abstract

This paper examines self-identified meaningful activities in the daily lives of 21 vulnerable older adults living with dementia and the people who care for them at home (dyads). Using ethnographic observation and interviews, we asked the dyads to identify which aspects of daily life were most meaningful and how these activities changed as dementia progressed. Results ranged from pleasure-seeking activities like cigarette smoking and eating, to spiritual or mindfulness activities like hymn-singing, prayer and tai chi. Dyads identified specific examples of the ways in which meaningful activities and meaning-making both persisted and adapted throughout the progression of dementia. Using these identifiable moments of meaning-making as a starting point for inquiry, we explore underlying questions of how to adapt to dementia progression while retaining meaning in relationships.

